# The phenomenology of mental imagery in people with intellectual disabilities

**DOI:** 10.1111/papt.12424

**Published:** 2022-08-26

**Authors:** Olivia Mary Hewitt, Peter E. Langdon, Susie A. Hales, Michael Larkin

**Affiliations:** ^1^ Centre for Educational Development Appraisal and Research (CEDAR) University of Warwick Coventry UK; ^2^ Learning Disability Services Berkshire Healthcare NHS Foundation Trust Reading UK; ^3^ Coventry and Warwickshire Partnership NHS Trust Rainbow Unit, Brooklands Hospital Birmingham; ^4^ Emotion and Mental Imagery Lab (EMIL) Department of Psychology Uppsala University Uppsala Sweden; ^5^ Department of Psychology Institute for Health and Neurodevelopment Aston University Birmingham UK

**Keywords:** intellectual disability, interpretative phenomenological analysis, learning disability, mental imagery, phenomenology, qualitative

## Abstract

**Objectives:**

Mental imagery is important in the development and maintenance of psychological disorders and well‐being but has been neglected in people with intellectual disabilities. A detailed idiographic analysis of the lived experience of mental imagery in this population is presented.

**Design:**

This qualitative study uses interpretative phenomenological analysis (IPA). It involved inclusive research methods with people with intellectual disabilities and other stakeholders (including family members, advocates, support workers and intellectual disability service managers).

**Methods:**

Ten individual semi‐structured interviews were conducted with people with mild–moderate intellectual disabilities. Participants were opportunistically sampled through organisations providing community services to people with intellectual disabilities in the UK. Two men and eight women (mean age 43 years) participated. Interviews were audio‐recorded and analysed using IPA.

**Results:**

People with intellectual disabilities are able to experience a range of rich and detailed mental images across all sensory modalities. Participants reported changes in affect based on mental imagery, and an ability to experience both spontaneous and deliberate mental images. The emotional saliency of the object of mental imagery appeared to influence participants' ability to engage with imagery. A number of adaptations make mental imagery more accessible and easier to report. The ability of people with intellectual disabilities to experience vivid mental imagery has important clinical implications for the use of a range of mental imagery interventions with this population.

**Conclusions:**

The need to consider mental imagery interventions for this population, and how these can be adapted to ensure accessibility is a priority for people with intellectual disabilities and psychological disorders.

**Practitioner points**

- People with intellectual disabilities can experience and engage with rich and vivid mental imagery across different sensory modalities.
- Specific techniques and strategies that facilitate or impede the use of mental imagery in people with intellectual disabilities are described.
- There are implications for using psychological interventions that incorporate mental imagery (e.g. imagery rescripting, generating positive images and metacognitive imagery‐based interventions) with people with intellectual disabilities.

## INTRODUCTION

Mental imagery refers to the internal mental processes whereby perceptual information is accessed from memory (rather than directly from external stimuli) and is described as ‘seeing with the mind's eye’ although it can be experienced across sensory modalities (Kosslyn et al., 2006). Distressing or intrusive mental imagery is associated with the development and maintenance of various psychological disorders (Hackmann et al., 2011) including post‐traumatic stress disorder and obsessive–compulsive disorder, and a lack of positive mental imagery is associated with depressive disorders (Holmes et al., 2016). Various mental imagery interventions have been developed to treat psychopathology, including for those with limited cognitive skills (e.g. Schwarz et al., 2020). People with intellectual disabilities experience higher rates of psychological distress than the general population (Cooper et al., 2007) and have limited access to evidence‐based psychological interventions, partly due to their cognitive abilities (Tsimopoulou et al., 2018).

Mental imagery abilities in people with intellectual disabilities are poorly understood. People with intellectual disabilities have difficulties with abstract thought, comprehending complex ideas and verbal and communication skills (British Psychological Society, 2015). Mental imagery is complex and disentangling potential difficulties in experiencing imagery from verbal and communication difficulties in conveying such experiences poses challenges. However, a systematic review suggests people with intellectual disabilities experience all aspects (i.e. the generation, maintenance, inspection and transformation) of mental imagery, although may require scaffolding to report their mental imagery (Hewitt et al., 2021). This review identified a dearth of studies around the phenomenology of mental imagery in this population. People with intellectual disabilities may experience less complex and vivid mental imagery (Brougham et al., 2020; Brown & Bullitis, 2006). Understanding how people with intellectual disabilities experience mental imagery has implications for the applicability of mental imagery interventions in this population.

Creating an image through drawing or collage, alongside verbal interviews, can elicit discussion and help researchers learn more about the phenomenon of interest (Gillies et al., 2005). People with intellectual disabilities can engage with qualitative research, including methods with a phenomenological stance (Rose et al., 2019). Whilst such research has been almost exclusive conducted solely through verbal interviews, Attard et al. (2017) note the constraints of language to convey the nuanced nature of some lived experiences, which is presumably amplified for people with intellectual disabilities. Integrating participant‐created drawings within semi‐structured interviews produced richer, deeper verbal data, with the images themselves providing useful information. Using self‐created images is an attempt to listen for the rich, aesthetic ‘inner dimension’ of the participants' words (Todres, 2007, in Attard et al., 2017), which provides an additional layer of meaning to be explored through interview and analysis. Images offer an additional information about the phenomenon and produce meanings independent of verbal material but can be used alongside verbal accounts to add depth and richness to our exploration of the phenomenon (Boden & Eatough, 2014). Therefore, visual and verbal data were gathered in the current study and analysed using interpretative phenomenological analysis (Boden et al., 2019) to explore mental imagery amongst people with intellectual disabilities.

### Aims of this study

This study used verbal methods (semi‐structured interview) and non‐verbal methods (creating an image) to explore the lived experience of mental imagery in people with intellectual disabilities.

## METHOD

### Approach and design

This qualitative study was designed and conducted from a phenomenological perspective. The project was developed via an inclusive research approach. This involved using co‐production with two separate groups, a group of people with intellectual disabilities (named the STAR Group), and a Stakeholder Group (comprising family members, advocates, support workers and intellectual disability service managers). Both groups were involved in the project conception, design and analysis. Whilst neither group were directly involved in writing this paper, due to the iterative nature of their input throughout the research process, their voice forms part of the authorial voice.

The semi‐structured interview schedule was co‐designed with the STAR and Stakeholder Groups. A range of topics were gathered (from the literature, a systematic review and discussion with clinicians working with mental imagery) and presented in accessible formats to the groups. Once topics for exploration in the interviews were agreed through group consensus, both groups were again consulted to discuss and agree experiential exercises to be included in the interview, and the format of the image participants would create. Adaptations to improve accessibility to the interview schedule were adopted.

Participants took part in one‐to‐one interviews. A favourable ethical opinion was given by a United Kingdom University Research Ethics Committee (Ref: 124/20–21). Informed consent was obtained from all participants.

### Sampling

Participants were recruited through opportunistic sampling from community settings specifically serving people with intellectual disabilities (e.g. day centres and self‐advocacy groups; Table 1). All participants were White British. Participants were excluded who did not have capacity to make a decision about whether they wished to take part in the study, were under 18 years of age, or had autism. Participants needed to be able to communicate verbally and describe their internal experiences of mental imagery.

**TABLE 1:** Participant demographic information

Pseudonym	Age	Gender	Ethnicity	Diagnoses	Living environment	Length of interview (minutes)
Clarissa	54	Female	White British	Mild ID	Residential home	54
June	43	Female	White British	Mild ID	Residential home	56
Bethany	48	Female	White British	Mild ID	Residential home	93
Peter	40	Male	White British	Mild ID	Shared lives placement	60
Anthony	48	Male	White British	Mild ID, dyspraxia	Family home	70
Zadie	46	Female	White British	Mild ID	Shared lives placement	51
Clemency	25	Female	White British	Mild‐to‐moderate ID	Supported living group home	48
Maria	30	Female	White British	Mild‐to‐moderate ID	Supported living group home	62
Karla	49	Female	White British	Mild‐to‐moderate ID, Down syndrome	Supported living group home	67
Rebecca	52	Female	White British	Mild ID, physical disability	Supported living group home	65

### Participants

The first author conducted individual interviews with 10 people with mild‐to‐moderate intellectual disabilities on one occasion. Eight women and two men aged 25–54 (*M*_age_ = 52.3 years) participated.

### Data collection

The semi‐structured interview had three phases:

Phase 1 explored the concept of mental imagery. A verbal description of mental imagery was provided. Participants were guided through an adapted mental imagery exercise to practice using mental imagery (Hackmann et al., 2011). Their mental imagery abilities and experiences were further explored.

During Phase 2, participants created an image, through drawing and/or collage, to reflect their experience of mental imagery. They could recreate an image which had already been explored during Phase 1 or choose a different image or memory to create using a selection of papers, art media (including pencils, biros, coloured pencils, felt‐tip markers, chalk pastels and acrylic paint), glues and a selection of magazines. Notes were taken on how participants approached the activity (Boden et al., 2019).

On completing their image, participants were guided to describe parts of the image, overall meaning, and the process of creating it, to illuminate the meaning of the image for the individual. Consideration was given to the anonymity of the images. Images were electronically copied, and participants kept the originals.

During Phase 3, the Vividness of Visual Imagery Questionnaire (Marks, 1973) was administered with prompts, to explore experiences of mental imagery.

Interviews lasted between 48 and 93 min (*M* = 62 min). Interviews were audio‐recorded and transcribed verbatim with pseudonymisation to ensure confidentiality.

### Data analysis

Transcripts and created images were analysed using interpretative phenomenological analysis (IPA; Smith et al., 2021). All co‐researchers contributed to each stage of data analysis (both verbal and visual materials), alongside the academic research team. OH completed initial verbal data analysis, involving each transcript being read repeatedly, exploratory notes being made detailing descriptive, linguistic and conceptual comments and experiential statements being compiled in each transcript, which were refined by seeking patterns and connections between them to develop super‐ordinate themes (Smith et al., 2021). Individual transcripts were revisited with these themes in mind, in an iterative process to ensure each participant's voice was captured. Visual and verbal data were treated similarly (with the participant's own meaning making as primary and with the researcher's interpretation also seen as important).

Data were presented to co‐researchers throughout this process in accessible formats including the use of post it notes, single words and symbols to represent aspects of the data, as well as portions of transcripts being role‐played. Visual images were similarly analysed using the meaning participants made of their image, combined with a structured framework for visual data analysis (Boden et al., 2019).

Data analysis took place in small groups in‐person and online and over multiple meetings. The Stakeholder Group met three times over 4 months (mean of eight attendees, range 7–9). The STAR Group met four times over 5 months (mean of seven attendees, range 4–13). Analysis was conducted during these meetings and during meetings with academic researchers, in an iterative process.

The STAR and Stakeholder Groups conducted all stages of analysis including recording initial impressions, identifying initial themes, making changes to the structure of superordinate and subordinate themes and choosing quotes to illustrate subthemes. The groups compared themes to find patterns across participants and develop the final set of themes. This was facilitated through using sticky notes to concretely represent themes from different participants which could be physically moved by group members. Discussions within the research team highlighted areas where the link between interpretative comments or themes and the raw data was less clear and allowed for consideration of how the researchers' own beliefs and preconceptions might influence analysis.

Quality assurance (Mays & Pope, 2020) was considered throughout this study. To ensure sensitivity to context, efforts were made during data collection to address the power imbalance between the researcher (OH) and participants with intellectual disability (e.g. starting with a brief general conversation to build rapport). To enhance rigour and transparency, an audit trail was documenting how themes developed for each participant and across participants was maintained. A reflective log was kept throughout the process.

## RESULTS

### Theme 1: Mental imagery involves making, comparing, checking and changing images

All participants could engage with and explore mental imagery (Table 2). The *clarity, detail* and richness of the images created and reported was notable. As well as creating mental images, participants described *changing* and manipulating these images. Participants could focus in or *check* details or *compare* aspects of an image. Most participants experienced several of these processes, suggesting an ability to manipulate and transform a *detailed*, malleable image, rather than experiencing flat, static mental images. Images could incorporate *movement* of objects, and participants seeing themselves *move* within an image. Such experiences conveyed a sense of imagery being under the participant's deliberate control.

**TABLE 2:** Outlines the themes and subthemes identified by the researchers

Superordinate themes	Subordinate themes
Theme 1: Mental imagery involves making, comparing, checking and changing images	Images can be clear and detailed
	Comparing different parts of an image
	Checking the details of an image
	Changing parts of an image
	Mental images can move
Theme 2: Our mental imagery can happen in any sense	Seeing mental images
	Touching mental images
	Hearing mental images
	Smelling mental images
	Tasting mental images
	Mental imagery can happen in more than one sense at the same time
Theme 3: Anything is possible in the world of mental imagery	Mental imagery can be surprising and funny
	Mental images can do impossible things vs mental images follow everyday rules
	We can share our mental images with other people
Theme 4: Mental imagery can have a big effect on our feelings	Mental imagery can give us strong feelings
	Mental imagery can make us feel lots of different feelings
	It can be easier or harder to imagine things that are important to us
Theme 5: Doing mental imagery can feel easy or difficult	Feeling like we're really good at mental imagery
	Sometimes mental imagery can feel too hard
	Asking us questions helps us have detailed mental imagery
	Helping us to concentrate makes mental imagery easier

Participants' mental imagery could be *detailed*, often including aspects of *movement* or being experienced in different sensory modalities. Peter spontaneously reported experiencing mental imagery in his everyday life, and during the interview he reported fluent, vivid and complex metal imagery without prompting:

I see the flowers, there's flowers and there's a big bird like a kite flying round and there's a tree beside me and it's summertime and it's really pretty, there's blue skies, the clouds, oh it's wonderful and it's relaxing to walk round. Oh, it's lovely, it's really lovely it makes me relax and makes me happy. It's really, really nice. I can see it as well. There's birds singing and it's wonderful. I can see rabbits and foxes.

Participants could ‘zoom in’ or focus on aspects of an image to *check* details, as June when inspected her image to report on the number of orange pips in a specific segment:

OH How many pips have you got there?

June Four.

Participants could hold an image in mind to *change* and *compare* aspects of it, which involved significant transformation of the image. Although June was asked to change the colour of her bedroom carpet, she went further than this, opting to change the material. She then used imagery to experience the appearance and texture of the wooden floor across sensory modalities. The speed and fluency with which she described the new floor indicated the richness and accessibility of her imagery as well as the ease with which she performed this transformation:

OH Imagine that green carpet that you've got… imagine that it changes colour.

June Instead of a carpet, get a wooden floor.

OH Yeah. Change that carpet to a wooden floor in your head, can you imagine that?

June Yeah.

OH Now when you were walking on that green carpet, it was feeling rough on your feet. Now you've got a wooden floor…

June Slippery!

The vividness of mental images included aspects of movement, as well as the ability to compare, change and check aspects within the image.

### Theme 2: Our mental imagery can happen in any sense

Almost all participants experienced mental imagery across different sensory modalities. Reporting mental imagery in the *visual modality* appeared to be the easiest and most common experience initially. However, most participants could, with practice and feedback, access and report mental imagery in other sensory modalities and, for several participants, *across several sensory modalities simultaneously*. This experience points to the phenomenology of mental imagery for participants; whilst it is experienced as distinct from perceptual experiences, it is also experienced ‘as if’ real. This was conveyed to the interviewer both verbally and through participants' facial expressions and body language.

Zadie had not experienced mental imagery before the interview but quickly understood what was meant by the term and subsequently *experienced mental imagery across different sensory modalities simultaneously*. When asked to imagine *tasting* an orange she declined to do so and instead, with the interviewer, built an alternative *visual* image of enjoying an ice cream at the beach. Several participants could use mental imagery flexibly, rather than simply following instructions from the interviewer. Zadie's ability to experience parts of the image as pleasurable (the *taste* of chocolate) whilst simultaneously finding other sensory aspects (the sticky *feeling)* unpleasant, and noting the *coldness* of the ice cream demonstrates the complexity and idiosyncratic nature of mental imagery:

OH This chocolate ice cream…

Zadie It's in a cone, with a chocolate flake and you lick it, and it goes down in the cone really fast.

OH And is it a bit melting this chocolate ice cream?

Zadie Yeah. And I've got it all round my face and hands.

OH How does it feel on your hands?

Zadie Sticky and horrible.

OH What does it taste like?

Zadie Oh, lovely!

OH And is it very cold the ice cream?

Zadie Very cold.

Participants could experience rich and detailed imagery when reporting several aspects of a single sensory modality. Bethany was able, with prompting, to report several aspects of the *tactile* experience of her image, including the weight, texture and temperature of an imagined orange:

OH What does it feel like?

Bethany Cold.

OH It feels cold, yeah.

Bethany You can peel it off and start eating it.

OH Yeah. Is it heavy or is it light, this orange?

Bethany Heavy.

OH It feels quite heavy. And what does the skin feel like, it feels cold, but is it smooth or bumpy?

Bethany Smooth.

These quotes illustrate the ability of participants to experience vivid imagery across all sensory modalities, including *smell*, as Bethany later described ‘[it] smells of orange… it is a nice smell’. The ability to experience an image across several sensory modalities simultaneously was notable, as was experiencing different aspects of one sensory modality, both of which provided a vividness to the image.

### Theme 3: Anything is possible in the world of mental imagery

This theme encompassed a range of experiences whereby images could be *surprising*, extraordinary and *humorous*. Whilst in theme one participants' mental imagery could be predictably transformed, remaining under their control, this theme included unbidden, unexpected aspects of imagery. Examples of this included Maria discovering her imagined orange was rotten ‘No, it's gone off, sorry… it's bad for you, it will give you an upset tummy’ and Peter spontaneously visualising ‘A big ball in my bedroom [laughs]… It's like all different colours… It's bouncing, bouncing, bouncing, bouncing all in my room’. The inclusion of movement in an image (often unexpected) gave a sense of the unpredictability of imagery, with it shifting and developing in different directions, seemingly only partially controlled by the participant.

A dichotomy of experiences emerged, whereby for some participants their *mental imagery abided closely by real life rules* (including their physical limitations and socially constructed rules around Covid restrictions). One participant with dyspraxia could not imagine catching an orange due to their physical limitations. Karla's *mental imagery abided by the real‐life rule* of wearing a face mask, preventing her from eating an orange: ‘I can't feel… I can't eat it with my mask’.

However, for several participants, *mental imagery could be used to do impossible things* such as journeying into space. Maria was especially interested in unicorns, as reflected in her created image and her interview. With little prompting, and a sense of this image unfolding in her mind's eye, she fluently described a magical nightscape:

OH Imagine the sun going down in the evening…

Maria It's shining and it's yellow and bright and the moon is out and all the stars are out and all the lovely stars are twinkling in the sky and the moon is full, a full moon and a happy moon, not a sad moon. And he's got his eyes shut, it's bedtime and the stars are out twinkling down and the sun is…and the sun is awake because it's night time and it's yellow due to the sunshine, and then that's it. And the stars have unicorns around it; all round the stars are unicorns.

For such participants mental imagery could be used to explore *impossible scenarios* and was not *restrained by the everyday*. They had a confidence and fluency around experiencing and reporting such imagery.

Several participants used noise and gestures to convey their mental imagery to the interviewer, acknowledging that whilst mental imagery is an intrinsically internal experience, it can also *be shared with others*:

OH What noise are the waves making on the beach?

Clarissa Shshshsh. Beautiful!

OH And it's quite breezy. And can you hear any seagulls?

Clarissa Yeah… [makes squawking noise]

OH And are the dogs playing in the water?

Clarissa Yeah, they're barking away.

This elicitation of imagery required a *shared*, relational space to be created between the interviewer and participant. Whilst imagery was generated within the participants internal world, the process of communicating with the other and being prompted by the ideas and suggestions of the interviewer, led to a collaborative and iterative process of further *sharing* and elaboration of imagery.

This theme encompasses the wide range of mental images experienced by participants. Whilst engaging with mental imagery was both personal and idiosyncratic, it could be shared with others and include playful, unexpected aspects.

#### Theme 4: Mental imagery can have a big effect on our feelings

Almost all participants described changes in affect linked to experiencing mental imagery. Changes in feelings could be *strong* and powerful and encompassed *a wide range of different emotions*, such as surprise, delight, disgust and fear. They were communicated both verbally and nonverbally (e.g. through changes in facial expressions, voice), which conveyed the *intensity* of these emotional responses. When experiencing mental imagery, participants could describe their *associated emotions*, and respond to emotional reactions ‘as if’ they occurred in real life (e.g. exploring details of pleasurable images or avoiding feared situations within mental images).

Zadie demonstrated *a range of emotional* and behavioural responses to mental imagery including exploring pleasurable images in different sensory modalities:

OH So let's imagine…on your bedside table there's a really big, lovely bunch of flowers.

Zadie Oh, that would be lovely! Red, yellow, pinks, oranges, all different colours.

OH What do they smell like?

Zadie Really, really nice, with a nice vase.

Conversely, other images prompted anxiety, and associated behaviour change:

Zadie There's gonna be a big thunderstorm… All the clouds come, they're getting closer and closer…and I'm a bit scared so I go to my room and hide under the duvet.

Participants' ability to engage with mental imagery around emotionally salient items varied in unpredictable ways. Participants found it *both easier or harder to imagine things that were important or held emotional significance*.

For some, focusing on a familiar and loved object, such as a pet, facilitated their ability to generate mental imagery. However, asking people to engage with mental imagery around other precious items could impede imagery as people protected these items from perceived harm or disrespect. For several participants, the *strength of affect* generated was noticeable. Clarissa spoke about her neighbour's cat Marley throughout her interview. She was very fond of the cat and generated, developed and transformed mental images of it. Subsequently, however, imagining Marley impeded her mental imagery. Marley was not allowed in Clarissa's house, and when asked to imagine Marley in her bedroom she had a *strong emotional response*:

Clarissa Ergh! That's horrible. I wouldn't allow it.

OH You don't like it do you? I can see from your face.

Clarissa She wouldn't be allowed upstairs. She wouldn't be allowed up there.

Another example of the impact of the emotional saliency of objects of the participant's ability to incorporate them into mental imagery occurred when Clarissa was asked to imagine herself jumping on her bed:

OH Get on the bed and start jumping up and down! Can you imagine?

Clarissa No!

OH Just imagine that in your head.

Clarissa It'll break it….

OH Can you imagine doing it?

Clarissa Nope.

OH Can you imagine me doing it?

Clarissa No!

It transpired the bed was a gift from Clarissa's parents and she was reluctant to treat this precious item disrespectfully.

Understanding the idiosyncratic links between the emotional saliency of an object and the impact this may have on a persons' ability to engage with mental imagery around it can be subtle, requiring careful exploration.

### Theme 5: Doing mental imagery can feel easy or difficult

Participants experienced a continuum of mastery over mental imagery. For some, mental imagery across different sensory modalities *was a pre‐existing skill they could effortlessly engage in*. For example, Peter frequently spontaneously reported complex mental imagery across sensory modalities in a fluent and eloquent manner, reflecting the ease with which he experienced and could report these phenomena. For others it was an untapped resource, they were initially unaware of but could quickly and easily engage with through practice and receiving feedback on the imagery exercises in phase one. However, for a few participants mental imagery remained challenging to engage with throughout the interview, and they required additional prompting and scaffolding to experience any mental imagery.

Even with various strategies to support participants' engagement, almost all participants found some aspect of *mental imagery was too hard* or inaccessible. Such aspects varied between participants, although several participants found transforming images more difficult than generating new images or inspecting or checking existing mental images. Zadie struggled to change an image of an orange into a frog:

OH Do you find it easy to do or hard?

Zadie Some of it's a bit hard.

OH Which bits have been hard?

Zadie Well, the one you said about the frog.

Various techniques facilitated the development of mental imagery. Reducing external, environmental distractions and *improving concentration* was helpful (e.g. suggesting participants close their eyes). *Asking both open and closed questions* helped participants reflect on their images and develop them further. Scaffolding, which included repeating back descriptions of images, encouraged elaboration. Whilst providing additional prompts and scaffolding generally helped all participants to provide more detailed imagery, it was essential to help some engage with and report mental imagery. Clemency's level of verbal fluency and cognitive skills were somewhat lower than other participants. Whilst she *struggled to describe and report her mental imagery* to others at points in the interview, she could generate imagery in different sensory modalities relatively easily:

OH What do the mountains look like?

Clemency They are white.

OH Why are they white?

Clemency Because it's got snow.

OH They are very big mountains.

Clemency Don't like snow!

OH You don't like snow?

Clemency Cold.

Whilst all participants could engage in mental imagery to some extent, the aspects of mental imagery they found easiest varied. For some this was related to the emotional saliency of an object, whilst others found particular aspects of manipulating images difficult. Other could access mental imagery in specific modalities more easily.

## DISCUSSION

A phenomenological approach was used to explore experiences of mental imagery in people with intellectual disabilities. Participants reported rich, detailed mental images, which could be surprising, spontaneous, elicit emotions and experienced across sensory modalities. Their experiences creating, comparing, checking and changing mental images map onto theoretical aspects of mental imagery generation, inspection, maintenance, transformation (Kosslyn et al., 2006) and manipulation (Pearson et al., 2013). The only unrepresented theoretical stage was image maintenance, which occurs at a neural level.

All participants could engage with and report some aspects of mental imagery. There were many similarities with experiences of mental imagery within the general population. Whilst Brougham et al. (2020) reported that adults with intellectual disabilities can generate a compassionate image similarly to those without intellectual disabilities, Brown and Bullitis (2006) found only half of people with intellectual disabilities clearly experienced mental imagery. The current study indicated that adults with mild–moderate intellectual disabilities experience vivid, detailed mental imagery.

Participant's ability to experience mental imagery simultaneously across several sensory modalities indicates the complexity and richness of mental imagery experienced. The multi‐sensory nature of mental imagery was prompted for and facilitated through the semi‐structured interview. Previous research suggested that whilst participants with intellectual disabilities commonly experience visual mental imagery, fewer than half experienced tactile, olfactory, gustatory or kinaesthetic imagery even when specifically prompted (Brown & Bullitis, 2006).

Mental imagery could be surprising for participants as well as the interviewer. Several participants did not recognise the concept of mental imagery and denied ever experiencing mental imagery including daydreams (as found by Brown & Bullitis, 2006). This is commonly found within the general and clinical populations and is why mental imagery should be specifically asked about at assessment (Hales et al., 2015), rather than relying on spontaneous self‐report. However, participants willingly attempted various mental imagery exercises and could engage with such tasks. They seemed both pleased and surprised and occasionally would check they had retained this skill after the interview. Mental imagery could be unexpected, as when mental images transformed spontaneously. Mental imagery is both unbidden and deliberately constructed in the general population (Pearson et al., 2015).

Several participants spontaneously shared their internal experience of mental imagery with the interviewer, through consulting with them about changing their image, and using physical (gesturing the shape of a rainbow) or auditory (making the noise of a seagull) cues. Participants shared their internal experiences with the interviewer to build rapport and used mental imagery as a relational tool.

Engaging with mental images elicited a range of powerful emotions including joy, disgust and fear, as found in the general population and people undergoing psychological interventions (Skottnik & Linden, 2019). Brown and Bullitis (2006) reported several participants declined to image actions such as climbing a stepladder (due to fear of heights), concluding that mental imagery elicits associated emotions in people with intellectual disabilities. Such findings are in line with Pearson et al. (2015) who found that visual mental imagery activates the same neural circuits as perceptual information, but in a weaker form.

The emotional saliency of items could both facilitate and impede a participant's engagement with mental imagery. Participants engaged more willingly with preferred objects, although not universally, as when a participant imagined a much‐loved cat going into her bedroom (unallowed) and experienced a visceral reaction of outrage. Understanding why and when participants engage with various imagery is essential, as it may be erroneously assumed that not engaging with imagery is due to cognitive difficulties. Mental imagery can be emotional (as in this example) or non‐emotional (e.g. mentally rotating an abstract figure) (O'Donnell et al., 2020). The lack of meaningful, emotionally salient stimuli used in mental imagery tasks comparing the performance of people with intellectual disabilities with age‐matched controls (e.g. Meneghetti et al., 2018) may partially account for poorer engagement and performance from people with intellectual disabilities.

The ease and mastery with which participants engaged in mental imagery existed on a continuum. Whilst all participants could engage in some mental imagery, most required scaffolding to build detail, in line with Brougham et al. (2020). However, a couple of participants who provided detailed mental imagery without prompting, reported frequent, spontaneous mental imagery in everyday life. For several participants, mental imagery was difficult to generate and explore, requiring frequent prompting. The general population experience a range of mental imagery abilities, with a small proportion experiencing aphantasia (no mental imagery) (Keogh & Pearson, 2018). Level of cognitive ability did not seem related to mastery over mental imagery, with some of the more cognitively able participants struggling with this.

Within the general population people can be reluctant to report mental imagery, for example fearing it to be a sign of ‘going mad’ (e.g. Hales et al., 2015). Such reticence is overcome through detailed explanation of mental imagery and developing the therapeutic relationship. That participants in this study attempted mental imagery willingly may be partly due to providing a clear explanation of mental imagery and additional time developing rapport within the interview.

There is little evidence that mental imagery in people with intellectual disabilities differs substantially from the general population, with a recent systematic review concluding that ‘people with intellectual disabilities have similar mental imagery to other people, although it may be less vivid or complex’. (Hewitt et al., 2021). This study suggests that with adaptations, the mental imagery reported by people with intellectual disabilities can be as vivid and accessible as in the general population. Therefore, adapted mental imagery interventions for psychological distress (such as imagery rescripting and positive image generation) should be considered for this population. Indeed, rich mental imagery is found in other groups with cognitive limitations such as very young children, (Burnett Heyes et al., 2013), making mental imagery‐based clinical therapies suitable in such populations.

Various strategies facilitated mental imagery. Participants needed to clearly understand the task presented. Co‐producing this project with people with intellectual disabilities and stakeholders helped ensure materials were accessible. Participants, especially those experiencing mental imagery for the first time, required feedback to know they were completing a task correctly. Providing prompts and questions helped participants scaffold and develop their answers and elicited richer imagery. Taking time to build rapport was essential in developing their willingness to engage with mental imagery exercises. The interviewer is a clinical psychologist with many years' experience of working with people with intellectual disabilities, which may have optimised participants' engagement. Minimising external distractions facilitated mental imagery, and whilst some participants found closing their eyes helpful (to aid concentration and reduce competing visual stimuli), this was difficult for some participants with physical disabilities. The individual areas of strength and skills regarding mental imagery required careful exploration with participants, using a curious, non‐judgemental stance to optimise participants' abilities to engage in mental imagery. Several participants found incorporating movement into mental imagery challenging. For others, aspects of imagery transformation (such as in an imagery rescripting exercise) was hard. Understanding which aspects of imagery challenge an individual will be important when exploring mental imagery interventions with people with intellectual disabilities, as is providing practice and feedback on different types of mental imagery exercises.

This study employed an inclusive research approach (Frankena et al., 2019). People with intellectual disabilities and (separately) stakeholders were involved throughout the project, except for data collection and disseminating findings (although a detailed dissemination plan has been made). All co‐researchers were paid for their time.

Whilst co‐researchers with intellectual disabilities are increasingly involved with qualitative data analysis (Di Lorito et al., 2018), there have been no reports of this with IPA data to our knowledge. Adaptations to make data analysis accessible included academic researchers identifying and role‐playing portions of interviews and asking co‐researchers for their reflections, reading out quotes, using plain English to describe themes and asking people to reflect on their position when engaging in analysis (Cluley et al., 2021), thus helping everyone to understand why they might notice something different in the data based on their own personal experiences (i.e. drawing out the researcher's interpretations and the meaning they make from this).

### Limitations

Only participants from a white British background were recruited, limiting the experiences captured. All participants had mild–moderate intellectual disabilities. Directly including participants with severe and profound intellectual disabilities in research is challenging (Maes et al., 2021) and compounded when studying internal cognitive processes such as mental imagery. The parents and carers of several people with severe and profound intellectual disability were part of the stakeholder group.

Theme one maps closely onto the Kosslyn et al.’s (2006) model of mental imagery. The first author's familiarity with this model will have inevitably influenced their analysis, as will their position as a clinical psychologist working therapeutically with people with intellectual disabilities to explore their psychological experiences. The link between themes and verbatim quotes was emphasised throughout analysis. Involving different members of the research team in the analysis (including people with intellectual disabilities and stakeholders) ensured analysis was embedded in the data. Using supervision and a reflective log has supported this.

### Implications

The ability of people with intellectual disabilities to experience and report complex mental imagery suggests that a range of mental imagery interventions may be appropriate for this population. However, a recent review of CBT interventions within this population found no use of mental imagery interventions (Hronis, 2021). Further work should identify, adapt and test mental imagery interventions for prevalent mental health conditions (e.g. anxiety) within this population.

Using meaningful stimuli in mental imagery tasks and developing good rapport with participants with intellectual disabilities is essential for engagement, in both clinical and research settings. Flexibility in the type of mental imagery intervention used, and how this is presented to people with intellectual disabilities will be key in promoting successful engagement.

## AUTHOR CONTRIBUTIONS

**Peter E. Langdon:** Conceptualization; supervision; writing – review and editing. **Susie A. Hales:** Conceptualization; methodology; supervision; writing – review and editing. **Michael Larkin:** Conceptualization; methodology; writing – review and editing.

## FUNDING INFORMATION

OH is supported by an NIHR Clinical Doctoral Fellowship (NIHR300501). The views expressed are those of the authors and not necessarily those of the NHS, the NIHR or the Department of Health.

## CONFLICT OF INTEREST

All authors declare no conflict of interest.

## Data Availability

The data that support the findings of this study are available on request from the corresponding author. The data are not publicly available due to privacy or ethical restrictions.

## References

[papt12424-bib-0001] Attard, A. , Larkin, M. , Boden, Z. , & Jackson, C. (2017). Understanding adaptation to first episode psychosis through the creation of images. Journal of Psychosocial Rehabilitation and Mental Health, 4(1), 73–88.

[papt12424-bib-0002] Boden, Z. , & Eatough, V. (2014). Understanding more fully: A multimodal hermeneutic‐phenomenological approach. Qualitative Research in Psychology, 11(2), 160–177.

[papt12424-bib-0003] Boden, Z. , Larkin, M. , & Iyer, M. (2019). Picturing ourselves in the world: Drawings, interpretative phenomenological analysis and the relational mapping interview. Qualitative Research in Psychology, 16(2), 218–236.

[papt12424-bib-0004] British Psychological Society . (2015). Guidance on the Assessment and Diagnosis of Intellectual Disabilities in Adulthood online . https://www.bps.org.uk/sites/www.bps.org.uk/files/Member%20Networks/Faculties/Intellectual%20Disabilities/Guidance%20on%20the%20Assessment%20and%20Diagnosis%20of%20Intellectual%20Disabilities%20in%20Adulthood.pdf

[papt12424-bib-0005] Brougham, L. , Pert, C. , & Jahoda, A. (2020). Exploring the ability of individuals with an intellectual disability to generate and use a compassionate image. Journal of Applied Research in Intellectual Disabilities, 33(6), 1296–1306. 32430986 10.1111/jar.12749

[papt12424-bib-0006] Brown, R. I. , & Bullitis, E. (2006). The process of mental imagery in persons with or without intellectual disability: An exploratory project. Journal on Developmental Disabilities, 12(1), 1–18.

[papt12424-bib-0007] Cluley, V. , Pilnick, A. , & Fyson, R. (2021). Improving the inclusivity and credibility of visual research: interpretive engagement as a route to including the voices of people with learning disabilities in analysis. Visual Studies, 36, 1–13.

[papt12424-bib-0008] Cooper, S. A. , Smiley, E. , Morrison, J. , Williamson, A. , & Allan, L. (2007). Mental ill‐health in adults with intellectual disabilities: prevalence and associated factors. The British Journal of Psychiatry, 190(1), 27–35. 17197653 10.1192/bjp.bp.106.022483

[papt12424-bib-0009] Di Lorito, C. , Bosco, A. , Birt, L. , & Hassiotis, A. (2018). Co‐research with adults with intellectual disability: A systematic review. Journal of Applied Research in Intellectual Disabilities, 31(5), 669–686. 29231285 10.1111/jar.12435

[papt12424-bib-0010] Frankena, T. K. , Naaldenberg, J. , Cardol, M. , Garcia Iriarte, E. , Buchner, T. , Brooker, K. , Embregts, P. , Joosa, E. , Crowther, F. , Schormans, A. F. , Schippers, A. , Walmsley, J. , O'Brien, P. , Linehan, C. , Northway, R. , van Schrojenstein Lantman‐de Valk, H. , & Leusink, G. (2019). A consensus statement on how to conduct inclusive health research. Journal of Intellectual Disability Research, 63(1), 1–11. 29642277 10.1111/jir.12486

[papt12424-bib-0011] Gillies, V. , Harden, A. , Johnson, K. , Reavey, P. , Strange, V. , & Willig, C. (2005). Painting pictures of embodied experience: The use of nonverbal data production for the study of embodiment. Qualitative Research in Psychology, 2(3), 199–212.

[papt12424-bib-0012] Hackmann, A. , Bennett‐Levy, J. , & Holmes, E. A. (2011). Oxford guide to imagery in cognitive therapy. Oxford University Press.

[papt12424-bib-0013] Hales, S. , Blackwell, S. E. , Di Simplicio, M. , Iyadurai, L. , Young, K. , & Holmes, E. A. (2015). Imagery‐based cognitive‐behavioral assessment. In G. P. Brown , & D. A. Clark (Eds.), Assessment in Cognitive Therapy (pp. 69–93). Guilford Press. 26334081

[papt12424-bib-0014] Hewitt, O. , Steel, C. , Hales, S. , Hayden, N. , Gundeslioglu, H. , Tapp, K. , & Langdon, P. (2021). A systematic review and narrative synthesis of mental imagery tasks in people with an intellectual disability: implications for psychological therapies . [Manuscript submitted for publication]. CEDAR, University of Warwick. 10.1016/j.cpr.2022.102178 35738164

[papt12424-bib-0015] Heyes, S. B. , Lau, J. Y. F. , & Holmes, E. A. (2013). Mental imagery, emotion and psychopathology across child and adolescent development. Developmental Cognitive Neuroscience, 5, 119–133. 23523985 10.1016/j.dcn.2013.02.004 PMC6987813

[papt12424-bib-0016] Holmes, E. A. , Blackwell, S. E. , Burnett Heyes, S. , Renner, F. , & Raes, F. (2016). Mental imagery in depression: Phenomenology, potential mechanisms, and treatment implications. Annual Review of Clinical Psychology, 12, 249–280. 10.1146/annurev-clinpsy-021815-092925 26772205

[papt12424-bib-0017] Hronis, A. (2021). Cognitive behaviour therapy for people with intellectual disabilities—How far have we come? International Journal of Cognitive Therapy, 14(1), 114–132.

[papt12424-bib-0018] Keogh, R. , & Pearson, J. (2018). The blind mind: No sensory visual imagery in aphantasia. Cortex, 105, 53–60. 29175093 10.1016/j.cortex.2017.10.012

[papt12424-bib-0019] Kosslyn, S. M. , Thompson, W. L. , & Ganis, G. (2006). The case for mental imagery. Oxford University Press.

[papt12424-bib-0020] Maes, B. , Nijs, S. , Vandesande, S. , Van Keer, I. , Arthur‐Kelly, M. , Dind, J. , Goldbart, J. , Petitpierre, G. , & Van der Putten, A. (2021). Looking back, looking forward: Methodological challenges and future directions in research on persons with profound intellectual and multiple disabilities. Journal of Applied Research in Intellectual Disabilities, 34(1), 250–262. 33073444 10.1111/jar.12803

[papt12424-bib-0021] Marks, D. F. (1973). Visual imagery differences in the recall of pictures. British Journal of Psychology, 64(1), 17–24. 4742442 10.1111/j.2044-8295.1973.tb01322.x

[papt12424-bib-0022] Mays, N. , & Pope, C. (2020). Quality in qualitative research. Qualitative Research in Health Care, 320, 50–52. 10.1136/bmj.320.7226.50 PMC1117321 10617534

[papt12424-bib-0023] Meneghetti, C. , Toffalini, E. , Carretti, B. , & Lanfranchi, S. (2018). Mental rotation ability and everyday‐life spatial activities in individuals with Down syndrome. Research in Developmental Disabilities, 72, 33–41. 29080484 10.1016/j.ridd.2017.10.019

[papt12424-bib-0024] O'Donnell, C. , Di Simplicio, M. , & Heyes, S. B. (2020). Hypomanic‐like experiences and spontaneous emotional mental imagery. Journal of Affective Disorders, 277, 742–746. 32919295 10.1016/j.jad.2020.08.003

[papt12424-bib-0025] Pearson, D. G. , Deeprose, C. , Wallace‐Hadrill, S. M. , Heyes, S. B. , & Holmes, E. A. (2013). Assessing mental imagery in clinical psychology: A review of imagery measures and a guiding framework. Clinical Psychology Review, 33(1), 1–23. 23123567 10.1016/j.cpr.2012.09.001 PMC3545187

[papt12424-bib-0026] Pearson, J. , Naselaris, T. , Holmes, E. A. , & Kosslyn, S. M. (2015). Mental imagery: Functional mechanisms and clinical applications. Trends in Cognitive Sciences, 19(10), 590–602. 26412097 10.1016/j.tics.2015.08.003 PMC4595480

[papt12424-bib-0027] Rose, J. , Malik, K. , Hirata, E. , Roughan, H. , Aston, K. , & Larkin, M. (2019). Is it possible to use interpretative phenomenological analysis in research with people who have intellectual disabilities? Journal of Applied Research in Intellectual Disabilities, 32(5), 1007–1017. 31033103 10.1111/jar.12605

[papt12424-bib-0028] Schwarz, S. , Grasmann, D. , Schreiber, F. , & Stangier, U. (2020). Mental imagery and its relevance for psychopathology and psychological treatment in children and adolescents: A systematic review. International Journal of Cognitive Therapy, 13, 1–25.

[papt12424-bib-0029] Skottnik, L. , & Linden, D. E. (2019). Mental imagery and brain regulation—new links between psychotherapy and neuroscience. Frontiers in Psychiatry, 10, 779. 31736799 10.3389/fpsyt.2019.00779 PMC6831624

[papt12424-bib-0030] Smith, J. A. , Flowers, P. , & Larkin, M. (2021). Interpretative phenomenological analysis: Theory, method and research. Sage.

[papt12424-bib-0555] Todres, L. (2007). Embodied enquiry: Phenomenological touchstones for research, psychotherapy and spirituality. Springer.

[papt12424-bib-0031] Tsimopoulou, I. , Kroese, B. , Unwin, G. , Azmi, S. , & Jones, C. (2018). A case series to examine whether people with learning disabilities can learn prerequisite skills for cognitive behavioural therapy. The Cognitive Behaviour Therapist, 11, E1. 10.1017/S1754470X1700023X

